# Supplementary material for: Fast response of cold ice-rich permafrost in northeast Siberia to a warming climate
Source: Nat Commun. 2020 May 4;11:2201. doi: 10.1038/s41467-020-15725-8 (PMC7198584; doi:10.1038/s41467-020-15725-8)
Supplement: Supplementary file 1 — Supplementary Information [file 41467_2020_15725_MOESM1_ESM.pdf]

Supplementary Information for

FAST RESPONSE OF COLD ICE-RICH PERMAFROST IN NORTHEAST SIBERIA TO A WARMING CLIMATE

by JAN NITZBON et al.

Supplementary Methods 1

Calculation of the NESAL carbon stocks. We used total organic carbon (TOC) data from a total of 984 soil samples from the study region as input for the statistical bootstrapping approach by Strauss et al.[1] in order to derive representative soil organic carbon pools for the different landscape types considered in the simulations (drained lake basins (LB), Holocene deposits(HD), Yedoma deposits (YD)). Scaling the organic carbon pools with the areas of the respective landscape types (see Table 1 in Methods), resulted in estimated pools of $24.7\,$GtC for LB, $63.0\,$GtC for HD, and $13.0\,$GtC for YD, giving a total NESAL carbon pool of $100.7\,$GtC. Note that these pools include both active layer and permafrost deposits. For illustration, the total carbon pool was converted to an atmospheric CO_2 concentration using the conversion factor of 0.471 by Battle[2] and an airborne fraction of 0.5.

Supplementary Methods 2

Details on the numerical model. The numerical model of ice-wedge terrain we used for our study was based on the model setup by Nitzbon et al.[3,4] While we adapted most of the model setup used in that study, we extended and modified the model, in order to represent more processes and to be applicable to further landscape types. The changes compared to the model version of Nitzbon et al.[3] are detailed in the subsequent sections. Those process implementations and parameter values which are not discussed in the subsequent sections were adopted as described by Nitzbon et al.[3] The model code and the settings to reproduce the numerical simulations of the present study are available from `https://doi.org/10.5281/zenodo.3648266`.

Implementation of lateral sediment transport. The calculation of lateral sediment fluxes was adapted from Roering et al.[5] and Plug and West[6], who proposed a law for the effective hillslope sediment transport (q^{sed}) as a combination of diffusive (q^{diff}) and advective (q^{adv}) processes. The diffusive part of the sediment transport is increasing linearly with the slope. In our tile-based model the slope between two adjacent tiles is obtained from the difference in soil surface elevations (a) and the lateral distance between the tiles (D). The diffusive sediment flux (in m^2 s^{-1}) between two tiles i and j is thus given as follows (see blue curve in Supplementary Figure 11):

$$q^{\text{diff}}_{i \leftarrow j} = K_{\text{eff}} \, \frac{a_j - a_i}{D_{ij}} \tag{1}$$

where K_{eff} (m^2 s^{-1}) is a sediment transport coefficient detailed below. The advective part of the sediment transport is negligible for small slope angles, but diverges when a critical slope α_{crit} is approached. It can be expresses in terms of the slope angle $\alpha = \arctan\left(\frac{a_j - a_i}{D_{ij}}\right)$ as follows (see red curve in Supplementary Figure 11):

$$q^{\text{adv}}_{i \leftarrow j} = K_{\text{eff}} \frac{a_j - a_i}{D_{ij}} \frac{\alpha^2}{\alpha^2_{\text{crit}} - \alpha^2} \tag{2}$$

The total sediment flux is obtained by summing up the diffusive and advective contributions (see yellow curve in Supplementary Figure 11):

$$q^{\text{sed}}_{i\leftarrow j} = q^{\text{diff}}_{i\leftarrow j} + q^{\text{adv}}_{i\leftarrow j} \tag{3}$$

$$= K_{\text{eff}} \frac{a_j - a_i}{D_{ij}} \frac{\alpha^2_{\text{crit}}}{\alpha^2_{\text{crit}} - \alpha^2} \tag{4}$$

$$= K_{\text{eff}} \frac{a_j - a_i}{D_{ij}} \frac{1}{1 - \left(\frac{\alpha}{\alpha_{\text{crit}}}\right)^2} \tag{5}$$

The volumetric sediment flux between two tiles (in $\text{m}^3\,\text{s}^{-1}$) is obtained by multiplying the fluxes q with the contact length of the interface between the two involved tiles (L_{ij}). The sediment deposition rate (in $\text{m}\,\text{s}^{-1}$) of tile i is obtained by dividing the actual flux by the area of the tile (A_i).

The effective sediment transport coefficient (K_{eff}, also referred to as "hillslope diffusivity", in $\text{m}^2\,\text{s}^{-1}$) is calculated as the reciprocal sum of the land (subaerial) and water (subaqueous) part of the vertical interface between the soil surface altitudes of the involved tiles (see Supplementary Figure 12 for an illustration). In the illustration and the equations below, a refers to the soil surface altitude, and s refers to the surface altitude including water bodies above the the soil level. K_{eff} is calculated as follows:

$$K_{\text{eff}} = \frac{\varphi_{\text{tot}}}{\frac{\varphi_{\text{water}}}{K_{\text{water}}} + \frac{\varphi_{\text{land}}}{K_{\text{land}}}} \quad \text{, where:} \tag{6}$$

$$\varphi_{\text{tot}} = |a_i - a_j| \; , \tag{7}$$

$$\varphi_{\text{land}} = \max\left[0, \max\left(a_i, a_j\right) - \min\left(s_i, s_j\right)\right] \; , \tag{8}$$

$$\varphi_{\text{water}} = \max\left[0, \min\left(\varphi_{\text{tot}}, \min\left(s_i, s_j\right) - \min\left(a_i, a_j\right)\right)\right] \; . \tag{9}$$

For our simulations we used $K_{\text{land}} = 3 \times 10^{-10}\,\text{m}^2\,\text{s}^{-1}$ and $K_{\text{water}} = 3 \times 10^{-8}\,\text{m}^2\,\text{s}^{-1}$.

According to Plug and West[6] the value of the critical slope angle (α_{crit}) differs for frozen and unfrozen sediment. For unfrozen conditions we adopted the proposed value of $\alpha_{\text{crit}} = 45°$ by Plug and West[6]. For frozen ground conditions the authors suggested a value of $\alpha_{\text{crit}} = 90°$, leading to substantially reduced sediment transport during freezing conditions. For simplicity, we set lateral

sediment transport to zero during freezing conditions. An overview of the parameter values used for the lateral sediment transport is provided in Table 2.

In CryoGrid 3, the lateral sediment fluxes are calculated at each lateral transport time step (Δt_{lat}) which was set to 3600 s in our simulations. The sediment flux was further differentiated into mineral and organic contributions, according to the mineral and organic fractions in the uppermost grid cell of the more elevated tile. The lateral sediment transport scheme thus conserves the total sediment amount as well as the amounts of minerals and organics.

We assumed a dynamic equilibrium between the diffusive hillslope transport and the heaving of the ground surface due to aggradation of ground ice. Hence we did not explicitly apply the diffusive sediment fluxes, but only considered the advective fluxes. These advective fluxes correspond to rapid mass movement ("mass-wasting" or "slumping") which is observable in permafrost environments at steep unfrozen slopes.

Note that our model does not take into account periglacial processes like forst-cracking, ground ice accumulation, and frost-creep, which control periglacial landscape evolution on timescales of thousands to millions of years[7,8]. On timescales tens to hundreds of years, other processes such as ground subsidence and slumping are dominating the surface geomorphology in ice- and organic-rich soils, particularly under warming climatic conditions.

Hydrological boundary conditions. In our simulations the hydrological conditions were controlled via a hypothetical external "water reservoir" at a fixed altitude a_{res}. While Nitzbon et al.[3] used a reservoir which allows fluxes in both directions, i.e. from the troughs into the reservoir and vice versa, for the present study we excluded fluxes from the reservoir into the troughs.

The reservoir hydraulic conductivity (K_{res}) factors in the distance (D_{res}) and contact length (L_{res}) to the reservoir. Assuming that the modelled system is surrounded by a circular reservoir at

distance D_{res} gives a contact length of $L_{\text{res}} = 2\pi D_{\text{res}}$, such that the distance cancels out:

$$K_{\text{res}} = K \frac{L_{\text{res}}}{D_{\text{res}}} = 2\pi K \tag{10}$$

where $K = 1 \cdot 10^{-5}\,\text{m}\,\text{s}^{-1}$ is the same subsurface hydraulic conductivity which is used for the lateral water fluxes between two tiles.

Snow redistribution. Snow was redistributed among the different tiles (polygon centres, polygon rims, and troughs), assuming a preferential accumulation of snow in topographic depressions. The maximum height of the snow pack was limited to $h^{\text{max}} = 0.4\,\text{m}$ relative to the tile with the highest topographic elevation (including soil and water surfaces).

The snow redistribution scheme differed slightly from the implementation used by Nitzbon et al.[3] We scaled the incoming solid precipitation based on the differences in the surface altitudes (including snow cover) of the tiles, similarly to the scheme employed by Aas et al.[9] As long as the snow height of a tile is below a threshold height which reflects the snow catch effect of the vegetation ($h^{\text{catch}} = 0.1\,\text{m}$), the respective tile receives the full solid precipitation input from the forcing data. The snow input of low-lying tiles is scaled up, if there are higher-elevated tiles whose snow cover exceeds the threshold height. The higher-elevated tiles whose snow height exceeds the threshold height do not receive snow input until the lower-lying tiles' snow heights are equal. The scaling approach takes into account the different areas of the tiles such that the total amount of incoming solid precipitation is conserved.

Surface topology of ice-wedge polygons. The lateral transport schemes for heat, snow, water, and sediment require topological relations between the different tiles (polygon centres, polygon rims, and troughs). For the total area of one polygonal structure we assumed the same value as Nitzbon et al.[3] ($A_{\text{tot}} = 140\,\text{m}^2$), which is based on mapping of polygonal tundra at a well-studied site within the study area[10]. The areas of each tile are calculated using their areal fractions (γ) for

which we also used the same values as Nitzbon et al.[3] (see Table 1):

$$A_{\mathrm{C}} = \gamma_{\mathrm{C}} A_{\mathrm{tot}} \tag{11}$$

$$A_{\mathrm{R}} = \gamma_{\mathrm{R}} A_{\mathrm{tot}} \tag{12}$$

$$A_{\mathrm{T}} = \gamma_{\mathrm{T}} A_{\mathrm{tot}} \tag{13}$$

In order to derive further topological relations between the tiles, we assumed a nested circular geometry in which the polygon centres are embedded into circularly shaped rims and troughs (see Supplementary Figure 10 for an illustration). The (outer) radii of the respective circular structures are obtained as follows:

$$r_{\mathrm{C}} = \sqrt{\frac{A_{\mathrm{C}}}{\pi}} \tag{14}$$

$$r_{\mathrm{R}} = \sqrt{\frac{A_{\mathrm{C}} + A_{\mathrm{R}}}{\pi}} \tag{15}$$

$$r_{\mathrm{T}} = \sqrt{\frac{A_{\mathrm{C}} + A_{\mathrm{R}} + A_{\mathrm{T}}}{\pi}} \tag{16}$$

The radii can be used to calculate the lateral distances between the centre and rim tiles (D_{CR}), and between the rim and the trough tiles (D_{RT}) as follows:

$$D_{\mathrm{CR}} = \frac{1}{2}(r_{\mathrm{C}} + r_{\mathrm{R}}) \tag{17}$$

$$D_{\mathrm{RT}} = r_{\mathrm{T}} - \frac{r_{\mathrm{R}}}{2} - \frac{r_{\mathrm{C}}}{2} \tag{18}$$

The contact lengths between the center and rim tiles (L_{CR}), and between the rim and trough tiles (L_{RT}) are the perimeters of the respective circular structures:

$$L_{\mathrm{CR}} = 2\pi r_{\mathrm{C}} \tag{19}$$

$$L_{\mathrm{RT}} = 2\pi r_{\mathrm{R}} \tag{20}$$

The values of all topological parameters described above are provided in Table 1.

Subsurface stratigraphies of ice-wedge polygons. While we partitioned ice-wedge terrain based on the surface microtopography of polygonal tundra into three tiles (centers, rims, troughs), the

deeper subsurface of ice-wedge terrain is essentially partitioned into two units, namely pure wedge ice ("wed"), and ice-rich sediment between the ice wedges ("sed")[1]. In an idealized polygonal tundra landscape the polygon centers are underlain by ice-rich sediment while there is pure wedge ice below the troughs (down to the maximum depth of the ice wedges). The rims of the polygons are underlain partly by sediment and partly by wedge ice, depending on the lateral dimensions of the ice wedges (Supplementary Figure 10). We assumed the volumetric ground ice content to be $\theta_{\mathrm{i}}^{\mathrm{wed}} = 0.95$ for pure wedge ice, and $\theta_{\mathrm{i}}^{\mathrm{sed}} = 0.65$ for ice-rich sediment, following Strauss et al.[1] The volumetric ground ice contents of the three tiles (C,R,T) were composed of these values follows (see 10):

$$\theta_{\mathrm{i,C}} = \theta_{\mathrm{i}}^{\mathrm{sed}} \tag{21}$$

$$\theta_{\mathrm{i,R}} = \frac{1}{\gamma_{\mathrm{R}}} \left((\gamma_{\mathrm{sed}} - \gamma_{\mathrm{C}})\theta_{\mathrm{i}}^{\mathrm{sed}} + (\gamma_{\mathrm{wed}} - \gamma_{\mathrm{T}})\theta_{\mathrm{i}}^{\mathrm{wed}} \right) \tag{22}$$

$$\theta_{\mathrm{i,T}} = \theta_{\mathrm{i}}^{\mathrm{wedge}} \tag{23}$$

where γ_{sed} is the overall fraction of ice-rich sediment. $\gamma_{\mathrm{wed}} = 1 - \gamma_{\mathrm{sed}}$ is the overall fraction of wedge ice in the subsurface. It corresponds to the wedge-ice volumes provided in Table 1 for the different landscape types (LB, HD, YD). Soil layers for which the ice content (θ_{i}) exceeded the "natural" porosity ($\phi_{\mathrm{nat}} = 0.55$) were considered by the model to contain excess ice. The thickness of the ice-rich deposits (i.e. the depth of the ice wedges) was based on published estimates on ice-wedge dimensions from the study area[1,11] and field experience. We assumed best-guess depths for the NESAL of 4.0 m for LB, 10.0 m for HD, and 20.0 m for YD (see Tables 1).

The organic an mineral fractions were based on soil samples from the study region as described in the Mehtods section of the main text. An overview of the soil stratigraphies, including the organic, mineral, and ice contents as well as soil textures is provided in Tables 3 to 5.

Supplementary Methods 3

Reference runs. We conducted reference runs to reflect the response of the subsurface to warming climatic conditions in a model setup which is comparable to the design of land surface schemes of ESMs. For this we used CryoGrid 3 with a one-dimensional representation of the subsurface, i.e. without multiple tiles among which lateral fluxes occur. Moreover, the soil stratigraphy did not contain any excess ice (see Table 6). All surface water was considered as runoff and thus removed from the system. All other processes (heat conduction, snow scheme, hydrology scheme), parameter values, and the forcing data were identical to the model runs with a tiled setup. The simulation period included a 50-year spin-up (10/1949–12/1999) and a 100-year comparison period (01/2000–12/2099). We conducted one run under the RCP4.5 scenario, and one run under the RCP8.5 scenario.

The maximum thaw depth (11-year running mean) and the hydrological regime of unfrozen ground are display in Supplementary Figure 6. Under both warming scenarios the maximum thaw depth increased significantly within the simulation period. However, permafrost remained stable beyond 2100, as no permanently unfrozen layer (talik) formed in the simulations. This is in agreement with projections from land surface models for the same region[12–15]. Overall, permafrost degradation (i.e., lowering of the permafrost table) was significantly smaller in the reference runs compared to the runs with a tiled setup, since the latter took into account the melting of excess ground ice as well as feedback processes through lateral fluxes of heat, water, snow, and sediment.

Supplementary Methods 4

Simulations for an ambitious mitigation scenario (RCP2.6). We conducted additional simulations for the ambitious mitigation scenario RCP2.6. Supplementary Figure 7 shows the results in terms of accumulated ground subsidence, maximum thaw depth, and subsurface hydrological conditions, in analogy to Figure 3 of the main text.

Except for YD under water-logged conditions there is no subsidence due to melting of excess ground ice. The maximum thaw depths as well as the subsurface hydrological conditions remain stable throughout the simulation period. Under water-logged conditions YD show an accumulated mean subsidence of about 0.4 m, associated with the formation of a shallow surface water body (Supplementary Figure 7 f). This is, however, small compared to the thaw lake depth of about 2 m under RCP4.5 and about 4 m under RCP8.5 by the end of the simulation period (Figure 3 i,l).

In summary, under the RCP2.6 warming scenario ice-rich permafrost in the NESAL is projected to remain largely stable. Ice-wedge melt and surface water body formation could, however occur in very ice-rich Yedoma deposits. It should further be noted, that the process of ice-wedge growth and thus ground-ice accumulation could play a more important role for permafrost stability under RCP2.6 compared to RCP4.5 and RCP8.5. However, it is not taken into account by the model used for this study, and is likely to be negligible on the time scales of interest.

Supplementary Notes 1

Terminology and definitions. The terminology in our article largely follows the definitions in the "Cryosphere Glossary" of the the National Snow and Ice Data Center[16] and Kokelj and Jorgenson[17]:

Ground ice: a general term referring to all types of ice contained in freezing and frozen ground.[16]

Massive ice: a comprehensive term used to describe large masses of ground ice, including ice wedges, pingo ice, buried ice and large ice lenses.[16]

Wedge ice: ice occurring in an ice wedge.[16]

Excess ice: the volume of ice in the ground which exceeds the total pore volume that the ground would have under natural unfrozen conditions.[16]

Ice-rich permafrost: permafrost containing excess ice.[16]

Thermokarst (process): refers to the suite of processes by which characteristic landforms result from the thawing of ice-rich permafrost or melting of massive ground ice.[17]

Thermokarst landforms: Landforms emerging form thermokarst activity. In continuous permafrost these comprise, e.g., thermokarst lakes (or thaw lakes), drained lake basins ("alas"), thermokarst mounds ("baidzharakhs"), thermokarst troughs, high-centred polygons, thermo-erosional gullies, retrogressive thaw slumps, and active-layer detachments. These landforms co-occur or interact with each other, giving rise to other landforms (e.g. drainage of a thermokarst lake through incision of a thermo-erosional gully).

Thermokarst-inducing processes (numerical models): We introduce this term to refer to the structures and processes which are needed for representing the initiation and evolution of thermokarst

and associated feedbacks in numerical permafrost models. These comprise, but are not limited to

- representation of excess ground ice and ground subsidence upon thawing of ice-rich permafrost (e.g., refs. [18,19])
- ponding of surface water and related thermal processes (e.g., refs. [19–21])
- spatially heterogeneous distribution of (excess) ground ice (e.g., refs. [3,9,20])
- micro- (meters to tens of meters) and meso-scale lateral fluxes of heat, water, snow (e.g., refs. [3,9,20–22])
- micro-scale lateral fluxes of sediment (slumping) (e.g., this study)

Thermokarst-inducing processes can cause permafrost thaw dynamics which are fundamentally different from the gradual top-down thawing of permafrost in ice-poor terrain.

Supplementary Notes 2

Dominant types of excess ground ice and associated degradation pathways. Our study specifically addresses permafrost degradation pathways which occur in ice-rich permafrost landscapes that are underlain by massive ice wedges, since these constitute the dominant type of excess ground ice in the lowlands of northeast Siberia (NESAL) [23,24]. Other types of massive ground ice such as buried glacier ice, buried lake ice, or buried snowpacks are not common in the study area [23]. Past landscape evolution throughout the Holocene has preconditioned the northeast Siberian lowlands with different cryostratigraphies, characterized by different depths and sizes of ice wedges (Supplementary Figure 1). Our modelling approach thus focuses on the representation of ice-wedge degradation at the micro-scale (individual polygon scale), taking into account different abundances of wedge ice in the subsurface. At the meso-scale (or landscape-scale) the degradation of ice wedges can lead to the emergence of larger-scale thaw features such as thaw lakes or thermo-erosional valleys. While our model setup does not explicitly represent the spatio-temporal dynamics of these features (e.g. shoreline erosion or drainage of thaw lakes), the thaw processes involved at the micro-scale are reflected by performing simulations under contrasting hydrological conditions (water-logged versus well-drained).

By restricting our analysis to lowlands, we a priori exclude certain thermokarst features that are characteristic to hillslopes, such as landslides or active layer detachments[17]. We further do not address degradation pathways of ice-rich permafrost which are very localized (e.g., retrogressive thaw slumps) or linear (e.g., coastal erosion). For example, Nitze et al.[25] estimated that between 1999 and 2014 a total area of $1.08\,\mathrm{km}^2$ was be affected by 140 retrogressive thaw slumps within a transect of about $43000\,\mathrm{km}^2$ which intersects with a large part of our study area. Even if thaw slumps would become more abundant in a warming climate, the total area directly affected would still be small compared to our estimated areas of ice-wedge terrain. Similarly, Günther et al.[26] estimated recent erosion rates of ice-rich coastline along the Laptev sea (about $1400\,\mathrm{km}$) to be in the order of $5\,\mathrm{m\,yr}^{-1}$. Assuming a more pessimistic value of $10\,\mathrm{m\,yr}^{-1}$ over a 100 year time-frame, would yield a total affected area of $1400\,\mathrm{km}^2$. Hence, the total areas and carbon pools affected by

these mass-wasting lateral erosion processes are small compared to the vast areas underlain by ice wedges which would be affected by spacious ice-wedge degradation (about $493000\,\mathrm{km}^2$). Overall, we are thus confident that our model approach captures the dominant pathways of permafrost degradation in the study region.

Supplementary Notes 3

Relations between cryostratigraphy, subsidence, permafrost thaw, and thawed organic carbon. Our simulations shed light on the interrelations between cryostratigraphy, ground subsidence, permafrost thaw, and the amount of thawed organic carbon in a warming climate. As indicated in main Figure 3 j, the amount of thawed permafrost (ΔP) is composed of the accumulated ground subsidence (ΔS) and the increase in thaw depth (ΔD_{AL}):

$$\Delta P = \Delta S + \Delta D_{\mathrm{AL}} \tag{24}$$

Moreover, there is an idealized linear relation between permafrost thaw (ΔP) and ground subsidence (ΔS) when assuming that thawing ice-rich soil layers (immediately) settle with a (constant) natural porosity (ϕ_{nat}), and that the excess ice ($\theta_{\mathrm{i}} - \phi_{\mathrm{nat}}$) is removed from the soil column. Under these assumptions the following relation holds for ΔP:

$$\Delta P = \Delta S \, \frac{1 - \phi_{\mathrm{nat}}}{\theta_{\mathrm{i}} - \phi_{\mathrm{nat}}} \tag{25}$$

Equating the right hand sides of equations (24) and (25), and solving for ΔD_{AL} gives:

$$\Delta D_{\mathrm{AL}} = \Delta S \, \frac{1 - \theta_{\mathrm{i}}}{\theta_{\mathrm{i}} - \phi_{\mathrm{nat}}} \tag{26}$$

Similarly, ΔP can be expressed in terms of ΔD_{AL}:

$$\Delta P = \Delta D_{\mathrm{AL}} \, \frac{1 - \phi_{\mathrm{nat}}}{1 - \theta_{\mathrm{i}}} \tag{27}$$

Supplementary Figure 8 a shows the simulated and idealized relations between ΔD_{AL} and ΔS over the entire simulation period for the three different landscape types (i.e., cryostratigraphies) and the two hydrological conditions (water-logged, well-drained) considered in this study. As the above-mentioned assumptions underlie the excess ice scheme of CryoGrid 3, the simulated trajectories follow the idealized dependencies fairly well. As expected, ΔS increases with the mean excess ice content (θ_{i}) of the landscape type. Also ΔD_{AL} by the end of the simulation period increases with θ_{i}, reflecting that for the RCP8.5 scenario the permafrost is not in equilibrium with

the warmer climate. Overall, ΔP by the end of the simulation period shows a strong dependence on the cryostratigraphy.

However, as the volumetric organic contents (θ_o) vary among the sediments of the different landscape types, the relation between the additional organic carbon subject to thawed conditions (ΔC) does not show the same dependence on ΔS as ΔD_AL (Supplementary Figure 8 b). Interestingly, the value of ΔC by the end of the simulation period shows little dependence on the landscape type, indicating that different organic contents between the landscapes counterbalance different increases in thaw depth.

Overall, these considerations highlight the importance of quantifying both the ground ice content (θ_i) and the natural porosity (ϕ_nat) in order to establish reliable relations between permafrost thaw, subsidence, thawed organic carbon, and the cryostratigraphy.

Supplementary Notes 4

Sensitivity analysis. To analyze the sensitivity of the results against parameter variations, we conducted four additional sets of simulations, in which single parameter values were changed from their default values. For each varied parameter, we conducted simulations for the Holocene Deposits (HD) stratigraphy, under constrasting hydrological conditions (water-logged versus well-drained) and two warming scenarios (RCP4.5 and RCP8.5). The results of these simulations in terms of accumulated ground subsidence and increae in thaw depth are shown in Supplementary Figure 13. Note that additional sensitivity analyses were carried out in a preceding study[3].

The strongest deviations from the default runs were found when reducing the snow density to $\rho_{\text{snow}} = 200\,\text{kg}\,\text{m}^{-3}$. The onset of ice-wedge degradation was shifted to the middle of the twenty-first century under well-drained and to the 2020s under water-logged conditions. Under RCP4.5 ice-wedge stabilization occured only under well-drained conditions, and a thaw lake formed under water-logged conditions, reaching a depth of about $2.5\,\text{m}$ by 2100. Under RCP8.5 subsidence rates compared to the default setting were substantially higher under well-drained conditions, and of similar magnitude (but temporally shifted) under water-logged conditions. These findings highlight the crucial role of snow characteristics for the stability of permafrost.

When reducing the sediment transport coefficients by a factor of three to $K_{\text{land}} = 1{\cdot}10^{-10}\,\text{m}\,\text{s}^{-1}$ and $K_{\text{water}} = 1 \cdot 10^{-8}\,\text{m}\,\text{s}^{-1}$, respectively, the results in terms of subsidence and maximum thaw depth did not change significantly, suggesting that the lateral sediment transport scheme is robust against such variations. However, if the transport coefficients were reduced by orders of magnitude, ice-wedges would not stabilize and the results would become unphysical. Conversely, increasing the transport coefficients to very high rates, would lead to fast flattening of the terrain, standing in contrast to observations.

Reducing the field capacity to $\theta_{\text{fc}} = 0.4$, has almost no effect on simulated subsidence rates and thaw depths. Only under RCP4.5 and water-logged conditions (Supplementary Figure 13 c)

ice-wedge degradation was delayed by about two decades relative to the default run. The fractions of saturated and unsaturated conditions prevailing in thawed ground were not substantially affected by the change in field capacity (not shown).

By reducing the natural porosity to $\phi_{\text{nat}} = 0.45$ [1] the fraction of ice which is treated as excess ice is increased by 0.1. Consequently, the simulated subsidence rates and thaw depths for the HD stratigraphy, are similar to the corresponding results for Yedoma Deposits (YD) and default parameters ($\phi_{\text{nat}} = 0.55$; see Figure 3 c,f,i,l). However, this underlines the importance of constraining both the absolute ice content (θ_{i}) and the natural porosity of the ice-bearing sediments (ϕ_{nat}) in order to make quantitative predictions of ice-rich permafrost thaw.

In summary, the results were found to be robust against variations in parameters related to the hydrology scheme (θ_{fc}) and the lateral sediment transport scheme (K_{land}, K_{water}). While the results were affected by changes to the snow properties (ρ_{snow}) and the excess ice (ϕ_{nat}), our default choice for these parameters can be considered conservative.

[1] The field capacity was simultaneously reduced to $\theta_{\text{fc}} = 0.4$ to fulfill the constraint $\theta_{\text{fc}} < \phi_{\text{nat}}$.

Supplementary Figures

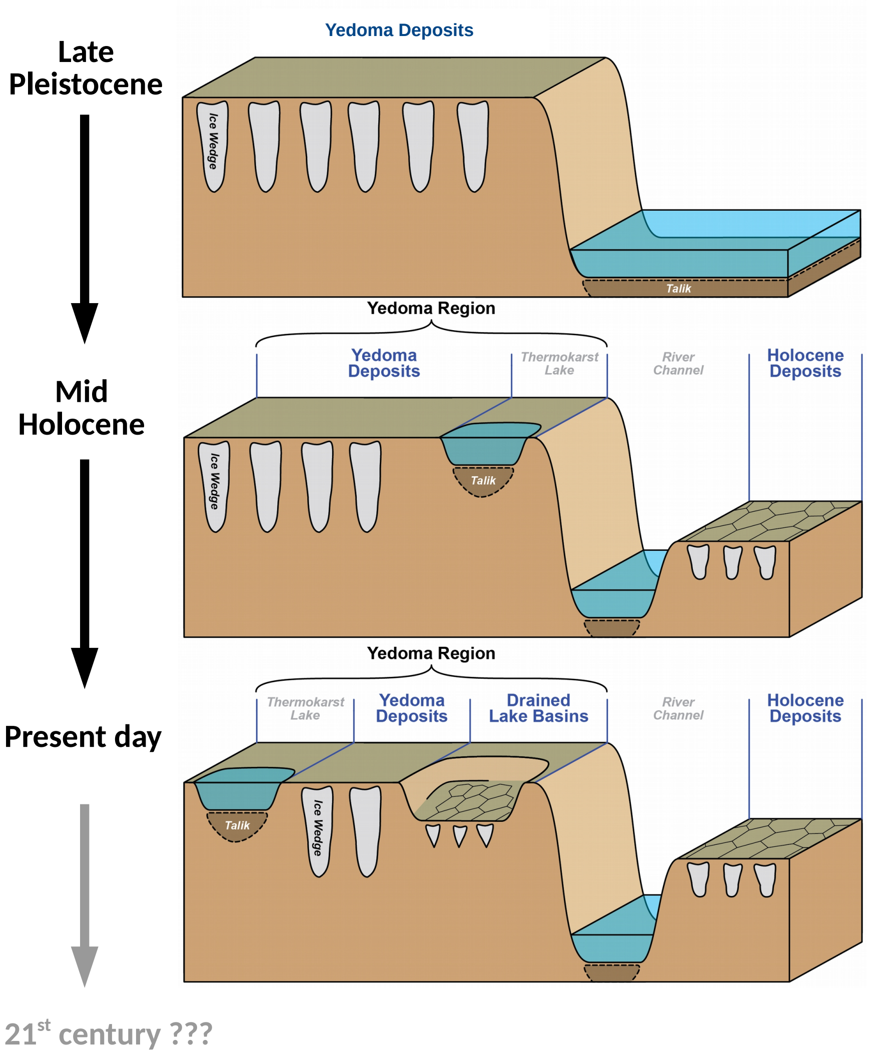

Supplementary Figure 1: Schematic evolution of ice-rich permafrost landscapes in the NESAL during the Holocene. Ice- and organic-rich Yedoma deposits which accumulated during the last glacial period started to degrade through thermokarst and thermoerosion processes under the warmer climate of the Holocene. Meanwhile the climate within the NESAL was cold enough such that ground ice accumulated in newly formed Holocene deposits, leading to the growth of so-called syngenetic ice wedges. Occasional drainage of thermokarst lakes led to permafrost aggradation in drained lake basins and the growth of so-called epigenetic ice wedges (conical shape). Depending on the evolution during the Holocene, different types of ice wedges underlie the NESAL at present. The distribution of ground ice preconditions the landscapes for different pathways of permafrost thaw in the future, for which a warming climate is projected.

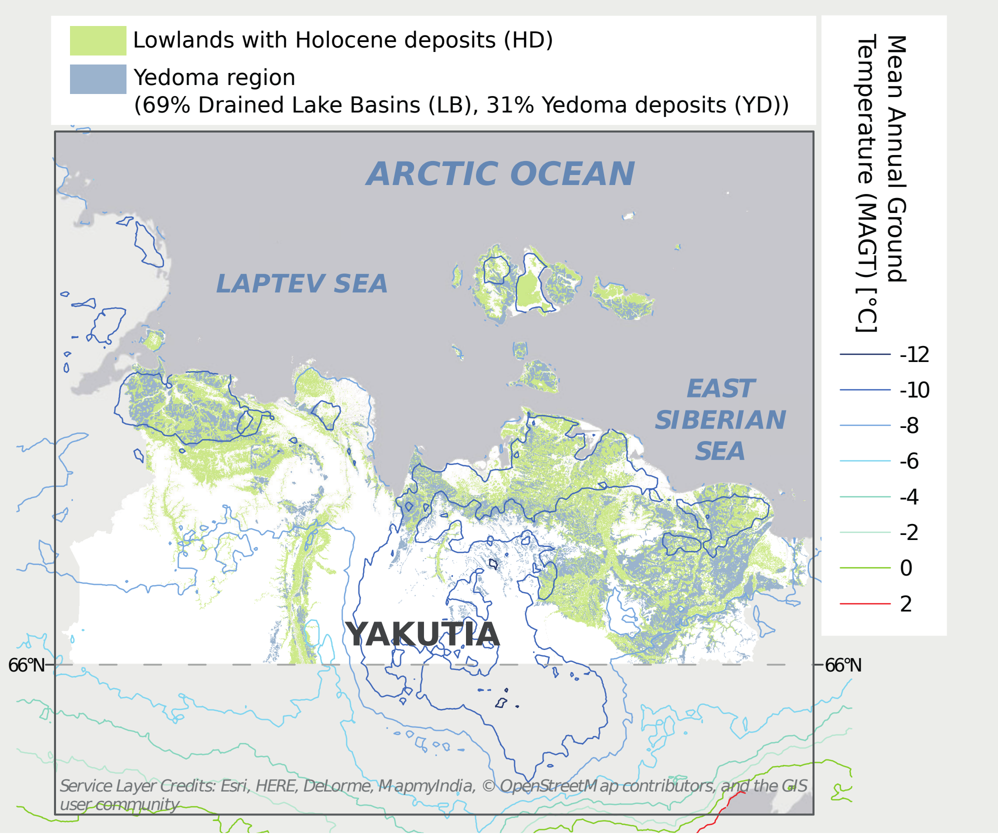

Supplementary Figure 2: Map of the northeast Siberian Arctic (white region). Lowlands belonging to the Yedoma region (undegraded Yedoma deposits (YD) and drained thermokarst lake basins (LB) are indicated in pale blue. Lowlands outside the Yedoma region with younger Holocene deposits (HD) are indicated in pale green. The majority of the lowlands has present-day mean annual ground temperatures ranging between -8 and -12°C. The Yedoma region was mapped by Strauss et al.[27]. Ground temperature data are taken from Obu et al.[28]

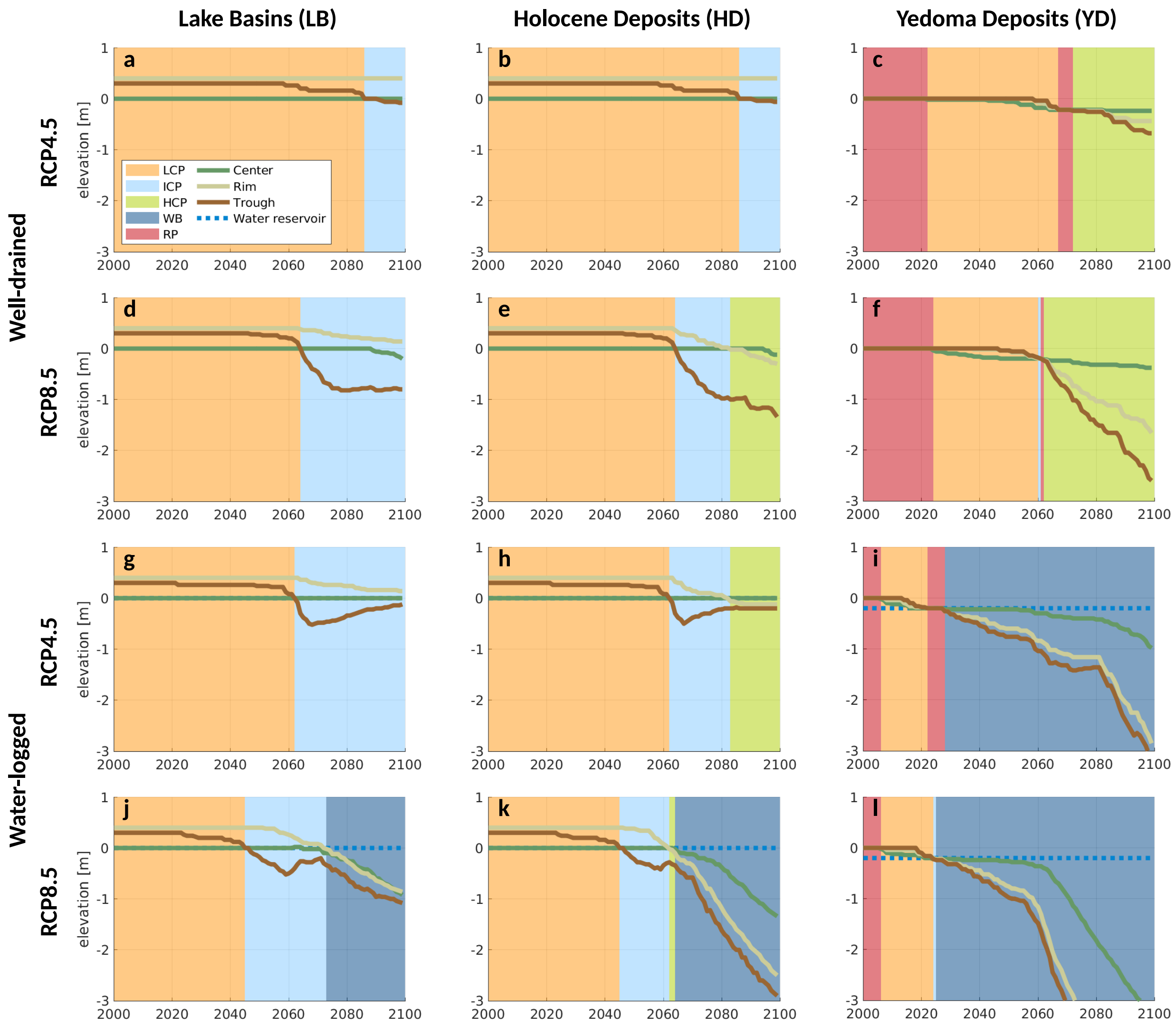

Supplementary Figure 3: Simulated evolution of the microtopography for all runs under RCP4.5 and RCP8.5. Bold lines show the soil surface altitudes of the three tiles (centres, rims, troughs), and background colours indicate the corresponding microtopographic states according to Eqn. (2) to (6).

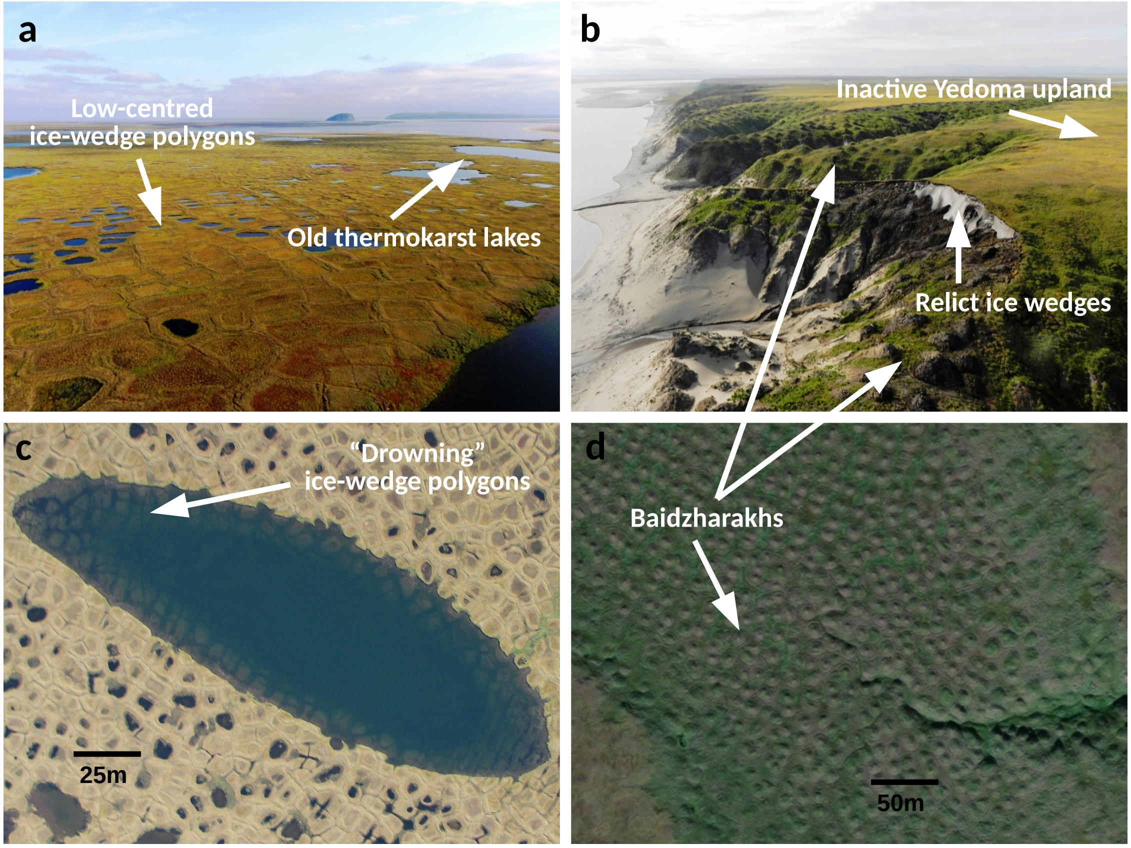

Supplementary Figure 4: Photographies of thaw features in the study region. The photographies show typical landforms and degradation features of ice-rich permafrost landscapes, corresponding to simulated states using our numerical model.

a: Ice-wedge polygons with a low-centred microtopography in the central Lena River delta (Samoylov Island). Small thermokarst lakes in the background are indicative of thermokarst activity in the past.

b: Yedoma deposits in the central Lena River delta (Kurungnakh Island). The undegraded uplands on the right have a flat surface topography while conical mounds termed ”Baidzharakhs” are visible in the center. These form through melting of relict ice wedges at the exposure.

c: Thermokarst lakes on Samoylov Island. The high-centred microtopography of the ice-wedge polygons is preserved at the bottom of the water bodies.

d: “Baidzharakhs” at the bottom of a drained lake basin on Kurungnakh Island.

Image credits: a,b: Sebastian Zubrzycki (distributed via imaggeo.egu.eu under a CC BY-NC-SA 3.0 license). c: The authors. d: Esri, DigitalGlobe, GeoEye, Earthstar Geographics, CNES/Airbus DS, USDA, USGS, AeroGRID, IGN, and the GIS User Community.

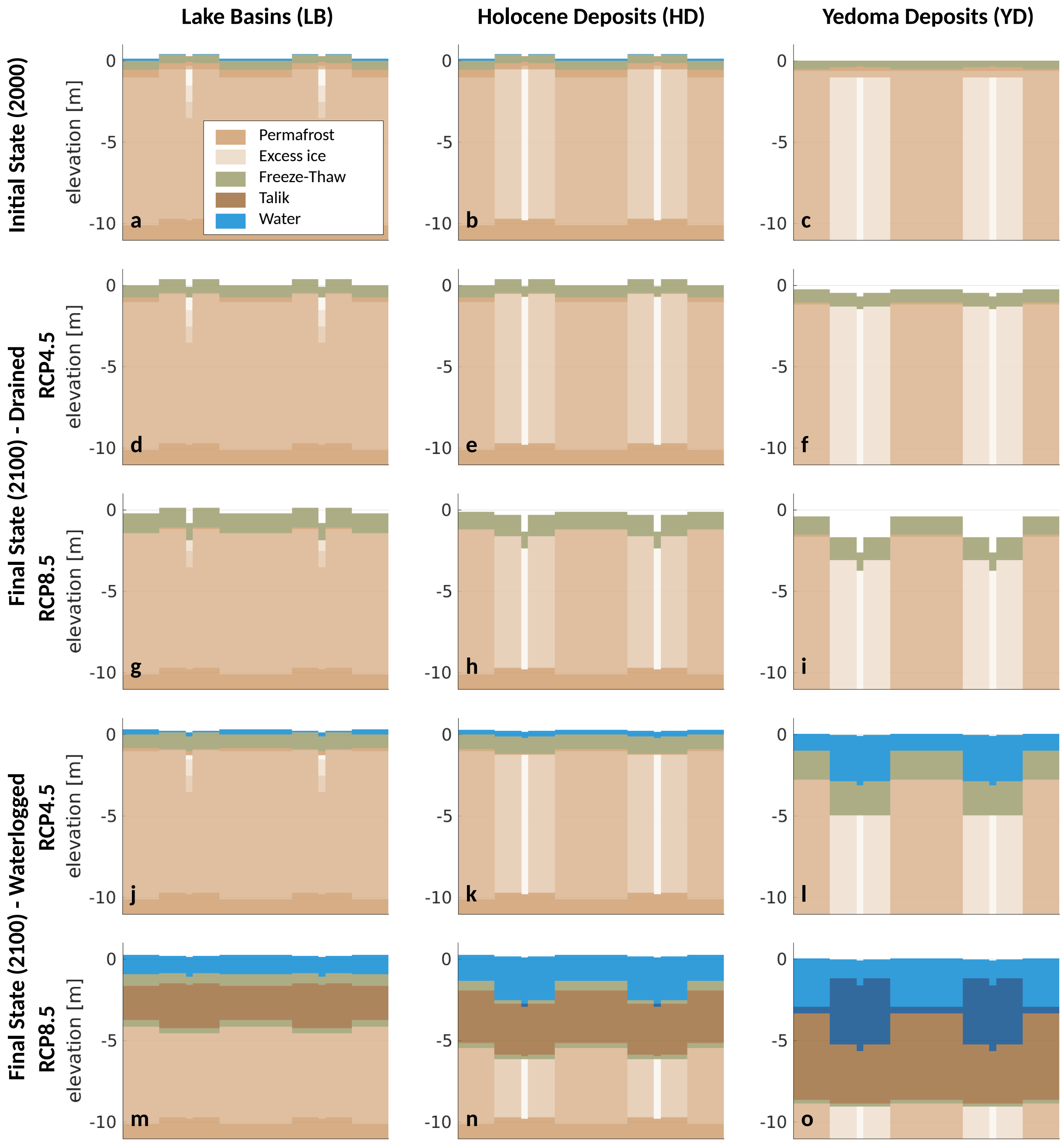

Supplementary Figure 5: Initial (2000) and final (2100) states of the landscape for all runs under RCP4.5 and RCP8.5. Layers containing excess ice are shown with a white overlay.

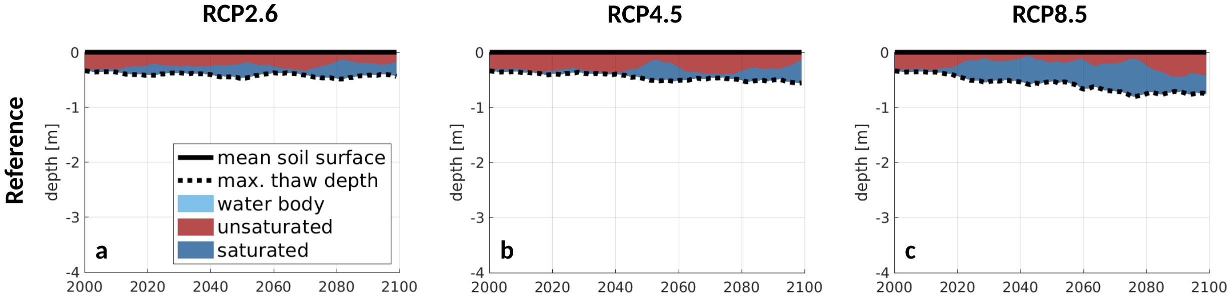

Supplementary Figure 6: Permafrost thaw in the reference runs. Dashed lines show the 11-year running mean of the maximum annual thaw depth for the reference runs without excess ground ice. As there is no ground subsidence (solid lines), permafrost degradation occurs only through active-layer deepening. Coloured areas indicate the fractions of unsaturated (red) and saturated (dark blue) conditions prevailing in the thawed ground throughout each year.

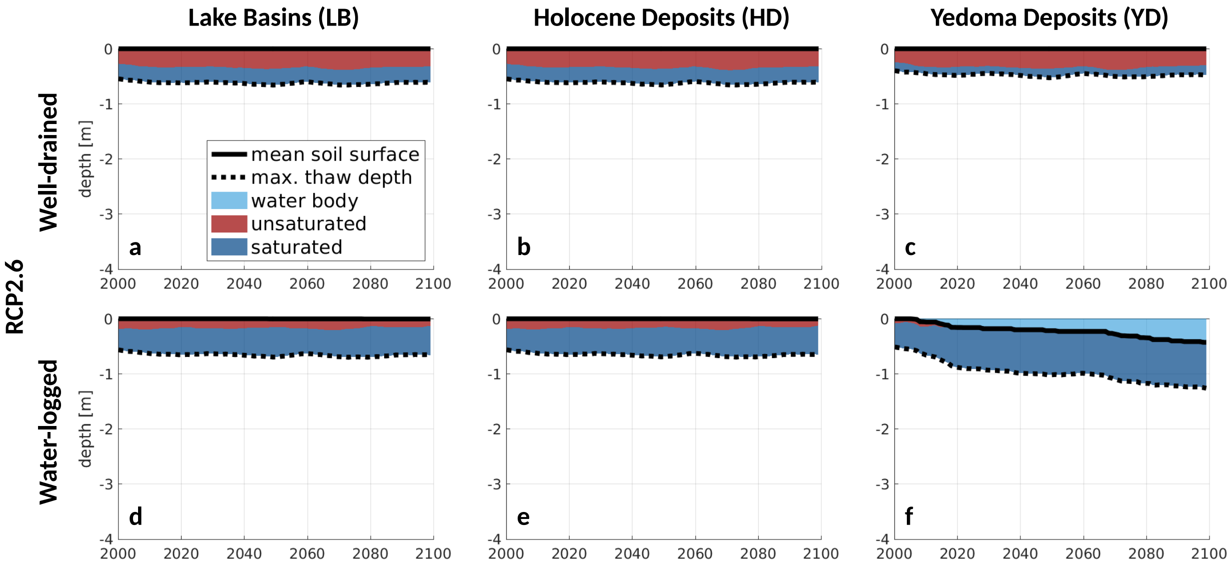

Supplementary Figure 7: Permafrost thaw in the RCP2.6 runs. Solid lines show the mean ground subsidence and dashed lines the 11-year running mean of the (area-weighted) maximum annual thaw depth for all runs under RCP2.6 warming scenario. Coloured areas indicate the fractions of unsaturated (red) and saturated (dark blue) conditions prevailing in the thawed ground throughout each year.

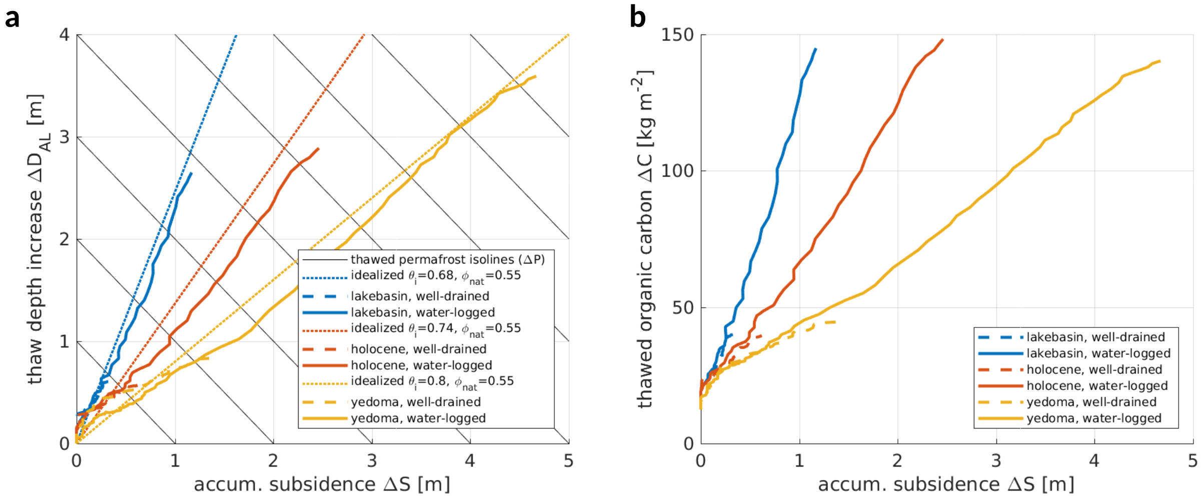

Supplementary Figure 8: Relations between the accumulated ground subsidence (ΔS) and the increase in maximum thaw depth (ΔD_{AL}, a), and the additional thawed organic carbon (ΔC, b). All displayed trajectories are for the RCP8.5 warming scenario.

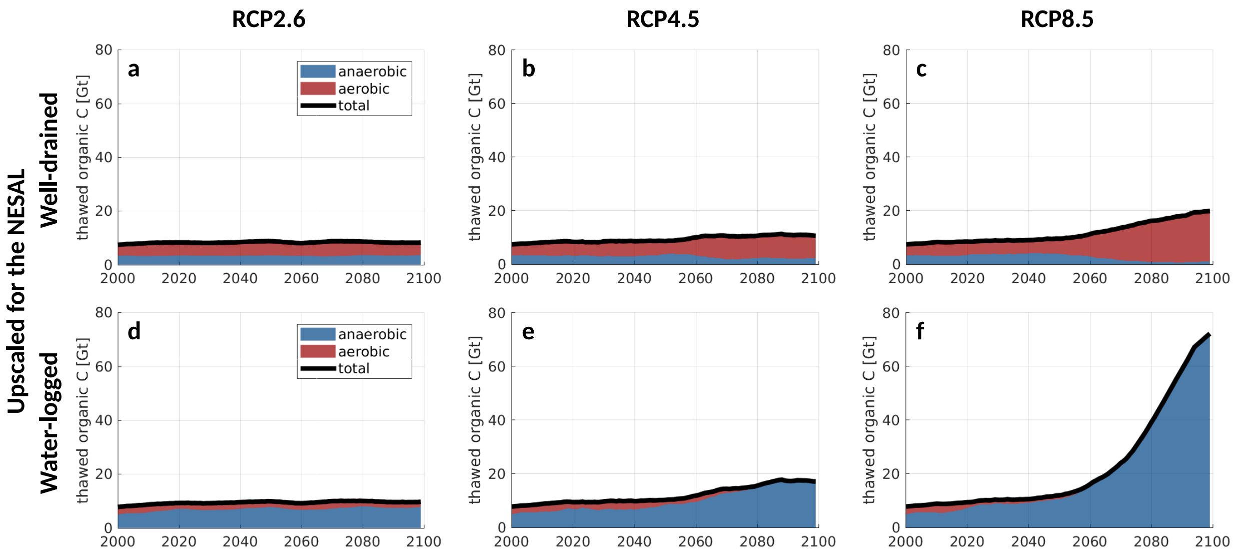

Supplementary Figure 9: Total amounts of thawed organic carbon from all landscape types within the NESAL for all combinations of hydrological conditions and warming scenarios. Coloured areas indicate the fractions of unsaturated (red) and saturated (dark blue) conditions to which thawed organic matter was exposed throughout each year.

Surface and subsurface partitioning

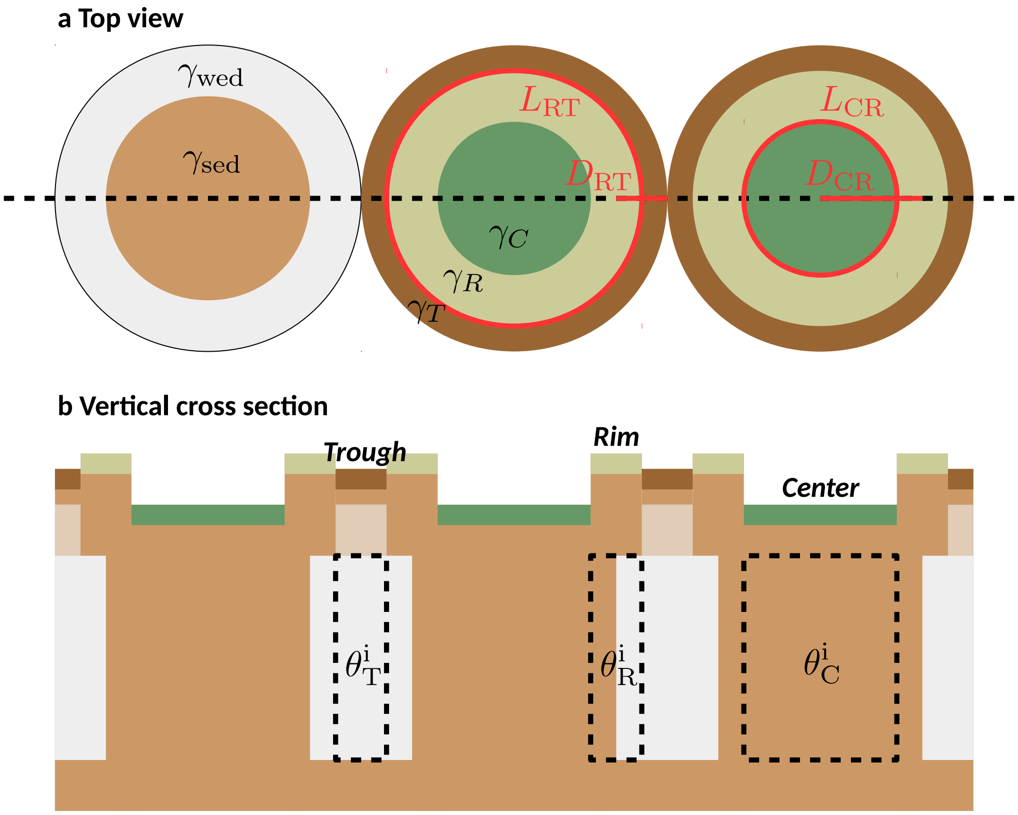

Supplementary Figure 10: Schematics of the partitioning of the surface according to microtopography and the subsurface according to ground ice distribution. The surface microtopography is divided into three tiles: polygon centers, polygon rims, and troughs. The subsurface is divided into wedge ice ("wed") and ice-rich sediment ("sed"). See text for definitions.

a: Topological relations between these tiles are derived under the assumption of nested circular shapes (a). The lateral distances (D) and contact lengths (L) are between the adjacent tiles are indicated in red.

b: While the subsurface of polygon centers is assumed to consist of ice-rich sediment, and the subsurface of troughs is assumed to consist of pure wedge ice, the subsurface of polygon rims is a mixture of the two, with the fractions $\gamma_{\rm wed}$ and $\gamma_{\rm sed}$ depending on the landscape type (LB, HD, YD).

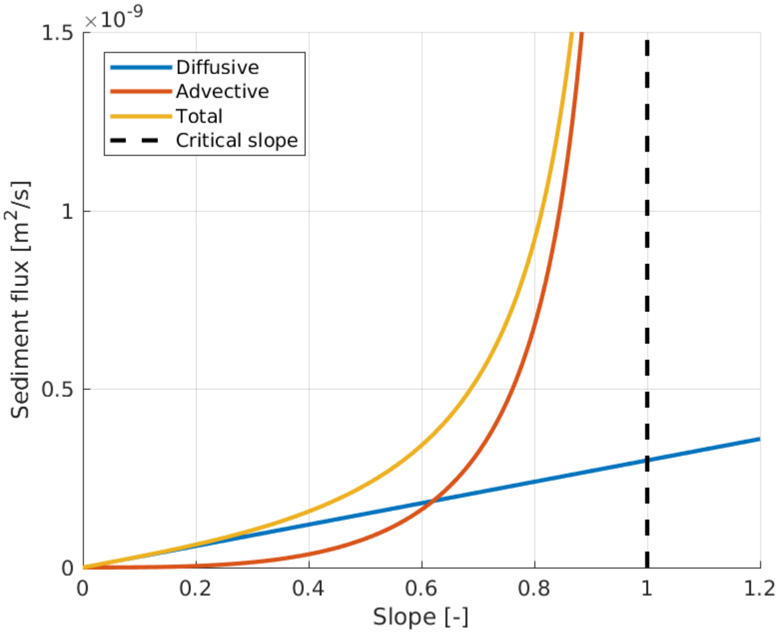

Supplementary Figure 11: Lateral sediment transport rates in dependence of slope. Diffusive, advective, and total lateral sediment flux rates between two tiles, according to equations (1), (2), and (5).

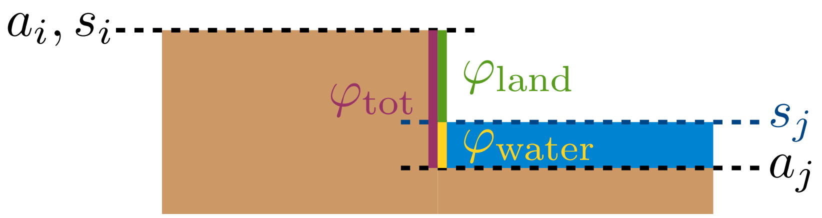

Supplementary Figure 12: Illustration of the lateral sediment transport coefficient. K_{eff} is composed of an subaerial and subaqueous parts.

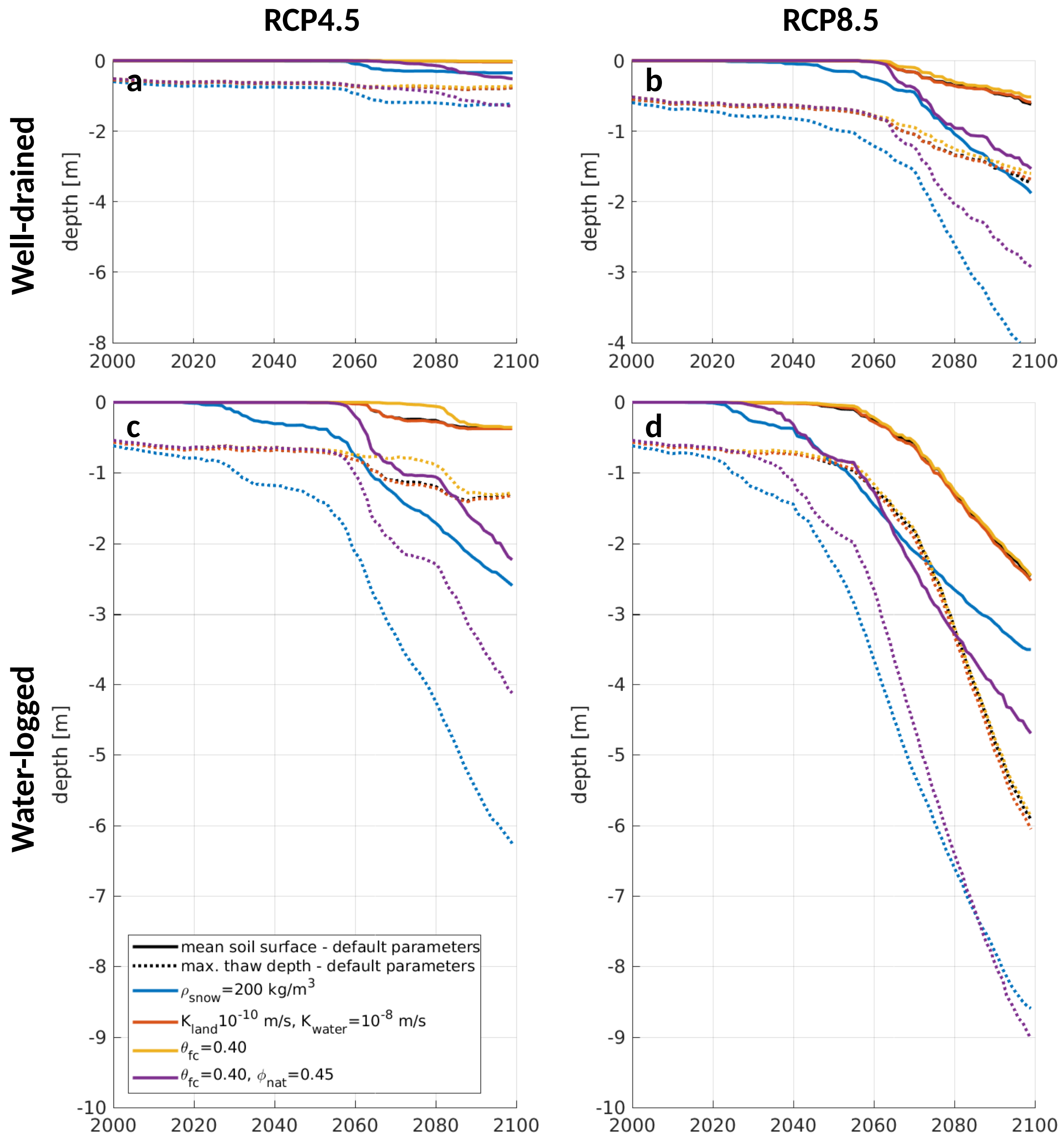

Supplementary Figure 13: Results of the sensitivity analysis. Each panel displays the accumulated ground subsidence and maximum thaw depth (11-year running mean) for simulations in which single parameters deviated from the default values. All results were obtained for the Holocene Deposits (HD) stratigraphy. See Supplementary Methods for a discussion.

Supplementary Tables

Supplementary Table 1: Parameter values reflecting the topology of ice-wedge polygons. Values for r, D, and L are rounded to one decimal place.

Parameter	Symbol	Unit	Tiles (α, $\alpha\beta$)				
			C	CR	R	RT	T
areal fraction	γ_α	-	0.3	-	0.6	-	0.1
area	A_α	m^2	42	-	84	-	14
outer radius	r_α	m	3.7	-	6.3	-	6.7
distance	$D_{\alpha\beta}$	m	-	5.0	-	1.7	-
contact length	$L_{\alpha\beta}$	m	-	23.0	-	39.8	-

Supplementary Table 2: Parameters of the lateral sediment transport scheme used for our simulations.

Parameter	Symbol	Unit	Value
Subaerial transport coefficient	K_{land}	$m^2\,s^{-1}$	3×10^{-10}
Subaqueous transport coefficient	K_{water}	$m^2\,s^{-1}$	3×10^{-8}
Critical slope angle	α_{crit}	$^\circ$	45
Lateral transport time step	Δt_{lat}	s	3600

Supplementary Table 3: Overview of the soil stratigraphies used for polygon centres, rims, and troughs of drained lake basins (LB). Excess ice layers are shown in bold. Depths are relative to the initial altitude (a) of the respective tile.

Depth [m]	Mineral θ_m	Organic θ_o	Nat. por. ϕ_nat	Soil type	Water θ_w^0	Comment
Lake Basins (LB)						
Center ($a_\mathrm{C} = 0.0\,\mathrm{m}$)						
0-0.1	0	0.15	0.85	sand	0.85	Vegetation layer
0.1-0.2	0.10	0.15	0.75	sand	0.75	Organic layer
0.2-1.0	0.25	0.10	0.65	silt	0.65	Mineral layer
**1.0-10.0**	**0.20**	**0.15**	**0.55**	**sand**	**0.65**	Ice-rich deposits
10.0–20.0	0.35	0.10	0.55	sand	0.55	Younger taberit deposits
20.0–40.0	0.50	0.05	0.45	sand	0.45	Older taberit deposits
>40.0	0.90	0	0.10	sand	0.10	Bedrock
Rim ($a_\mathrm{R} = 0.4\,\mathrm{m}$)						
0.0-0.1	0.10	0.15	0.75	sand	0.75	Organic layer
0.1-0.9	0.25	0.10	0.65	silt	0.65	Mineral layer
**0.9-10.0**	**0.20**	**0.15**	**0.55**	**sand**	**0.65**	Ice-rich deposits
10.0–20.0	0.35	0.10	0.55	sand	0.55	Younger taberit deposits
20.0–40.0	0.50	0.05	0.45	sand	0.45	Older taberit deposits
>40.0	0.90	0	0.10	sand	0.10	Bedrock
Trough ($a_\mathrm{T} = 0.3\,\mathrm{m}$)						
0-0.1	0	0.15	0.85	sand	0.85	Vegetation layer
0.1-0.2	0.10	0.15	0.75	sand	0.75	Organic layer
0.2-0.6	0.25	0.10	0.65	silt	0.65	Mineral layer
**0.6-0.8**	**0.20**	**0.15**	**0.55**	**sand**	**0.65**	Intermediate layer
**0.8-1.8**	**0.05**	**0**	**0.55**	**sand**	**0.95**	Ice wedge
**1.8-2.8**	**0.10**	**0.05**	**0.55**	**sand**	**0.85**	Ice wedge and ice-rich deposits
**2.8-3.8**	**0.15**	**0.10**	**0.55**	**sand**	**0.75**	Ice wedge and ice-rich deposits
**3.8-10.0**	**0.20**	**0.15**	**0.55**	**sand**	**0.65**	Ice-rich deposits
10.0–20.0	0.35	0.10	0.55	sand	0.55	Younger taberit deposits
20.0–40.0	0.50	0.05	0.45	sand	0.45	Older taberit deposits
>40.0	0.90	0	0.10	sand	0.10	Bedrock

Supplementary Table 4: Overview of the soil stratigraphies used for polygon centres, rims, and troughs of Holocene deposits (HD). Excess ice layers are shown in bold. Depths are relative to the initial altitude (a) of the respective tile.

Depth [m]	Mineral θ_m	Organic θ_o	Nat. por. ϕ_{nat}	Soil type	Water θ_w^0	Comment
Holocene Deposits (HD)						
Center ($a_C = 0.0\,m$)						
0-0.1	0	0.15	0.85	sand	0.85	Vegetation layer
0.1-0.2	0.10	0.15	0.75	sand	0.75	Organic layer
0.2-1.0	0.25	0.10	0.65	silt	0.65	Mineral layer
**1.0-10.0**	**0.20**	**0.15**	**0.55**	**sand**	**0.65**	Ice-rich deposits
10.0–30.0	0.50	0.05	0.45	sand	0.45	Older taberit deposits
>30.0	0.90	0	0.10	sand	0.10	Bedrock
Rim ($a_R = 0.4\,m$)						
0.0-0.1	0.10	0.15	0.75	sand	0.75	Organic layer
0.1-0.9	0.25	0.10	0.65	silt	0.65	Mineral layer
**0.9-10.0**	**0.15**	**0.10**	**0.55**	**sand**	**0.75**	Ice wedge and ice-rich deposits
10.0–30.0	0.50	0.05	0.45	sand	0.45	Older taberit deposits
>30.0	0.90	0	0.10	sand	0.10	Bedrock
Trough ($a_T = 0.3\,m$)						
0-0.1	0	0.15	0.85	sand	0.85	Vegetation layer
0.1-0.2	0.10	0.15	0.75	sand	0.75	Organic layer
0.2-0.6	0.25	0.10	0.65	silt	0.65	Mineral layer
**0.6-0.8**	**0.20**	**0.15**	**0.55**	**sand**	**0.65**	Intermediate layer
**0.8-10.0**	**0.05**	**0**	**0.55**	**sand**	**0.95**	Ice wedge
10.0–30.0	0.50	0.05	0.45	sand	0.45	Older taberit deposits
>30.0	0.90	0	0.10	sand	0.10	Bedrock

Supplementary Table 5: Overview of the soil stratigraphies used for polygon centres, rims, and troughs of Yedoma deposits (YD). Excess ice layers are shown in bold. Depths are relative to the initial altitude (a) of the respective tile.

Depth [m]	Mineral θ_m	Organic θ_o	Nat. por. ϕ_nat	Soil type	Water θ_w^0	Comment
Yedoma Deposits (YD)						
Center ($a_\mathrm{C} = 0.0\,\mathrm{m}$)						
0-0.1	0	0.15	0.85	sand	0.85	Vegetation layer
0.1-0.2	0.10	0.15	0.75	sand	0.75	Organic layer
0.2-0.6	0.25	0.10	0.65	silt	0.65	Mineral layer
**0.6-20.0**	**0.25**	**0.10**	**0.55**	**sand**	**0.65**	Ice-rich deposits
20.0–30.0	0.35	0.10	0.55	sand	0.55	Younger taberit deposits
30.0–50.0	0.50	0.05	0.45	sand	0.45	Older taberit deposits
>50.0	0.90	0	0.10	sand	0.10	Bedrock
Rim ($a_\mathrm{R} = 0.0\,\mathrm{m}$)						
0-0.1	0	0.15	0.85	sand	0.85	Vegetation layer
0.1-0.2	0.10	0.15	0.75	sand	0.75	Organic layer
0.2-0.6	0.25	0.10	0.65	silt	0.65	Mineral layer
**0.6-1.0**	**0.25**	**0.10**	**0.55**	**sand**	**0.65**	Intermediate layer
**1.0-20.0**	**0.10**	**0.05**	**0.55**	**sand**	**0.85**	Ice wedge and ice-rich deposits
20.0–30.0	0.35	0.10	0.55	sand	0.55	Younger taberit deposits
30.0–50.0	0.50	0.05	0.45	sand	0.45	Older taberit deposits
>50.0	0.90	0	0.10	sand	0.10	Bedrock
Trough ($a_\mathrm{T} = 0.0\,\mathrm{m}$)						
0-0.1	0	0.15	0.85	sand	0.85	Vegetation layer
0.1-0.2	0.10	0.15	0.75	sand	0.75	Organic layer
0.2-0.6	0.25	0.10	0.65	silt	0.65	Mineral layer
**0.6-1.0**	**0.25**	**0.10**	**0.55**	**sand**	**0.65**	Intermediate layer
**1.0-20.0**	**0.05**	**0**	**0.55**	**sand**	**0.95**	Ice wedge
20.0–30.0	0.35	0.10	0.55	sand	0.55	Younger taberit deposits
30.0–50.0	0.50	0.05	0.45	sand	0.45	Older taberit deposits
>50.0	0.90	0	0.10	sand	0.10	Bedrock

Supplementary Table 6: Overview of the soil stratigraphies used for the reference runs. Note that there are no excess ice layers. Depths are relative to the soil surface.

Depth [m]	Mineral θ_m	Organic θ_o	Nat. por. ϕ_nat	Soil type	Water θ_w^0	Comment
Reference run						
0-0.1	0	0.15	0.85	sand	0.85	Vegetation layer
0.1-0.2	0.10	0.15	0.75	sand	0.75	Organic layer
0.2-20.0	0.25	0.10	0.65	silt	0.65	Mineral layer
20.0–30.0	0.35	0.10	0.55	sand	0.55	Younger taberit deposits
30.0–50.0	0.50	0.05	0.45	sand	0.45	Older taberit deposits
>50.0	0.90	0	0.10	sand	0.10	Bedrock

Supplementary References

1. Strauss, J. *et al.* The deep permafrost carbon pool of the Yedoma region in Siberia and Alaska. *Geophysical Research Letters* **40**, 6165–6170 (2013).

2. Battle, M. Global Carbon Sinks and Their Variability Inferred from Atmospheric O2 and 13c. *Science* **287**, 2467–2470 (2000).

3. Nitzbon, J. *et al.* Pathways of ice-wedge degradation in polygonal tundra under different hydrological conditions. *The Cryosphere* **13**, 1089–1123 (2019).

4. Nitzbon, J., Langer, M., Westermann, S. & Martin, L. CryoGrid/CryoGrid3: CryoGrid 3 set-up for ice-wedge polygons (2019).

5. Roering, J. J., Kirchner, J. W. & Dietrich, W. E. Hillslope evolution by nonlinear, slope-dependent transport: Steady state morphology and equilibrium adjustment timescales. *Journal of Geophysical Research: Solid Earth* **106**, 16499–16513 (2001).

6. Plug, L. J. & West, J. J. Thaw lake expansion in a two-dimensional coupled model of heat transfer, thaw subsidence, and mass movement. *Journal of Geophysical Research: Earth Surface* **114** (2009).

7. Egholm, D. L., Andersen, J. L., Knudsen, M. F., Jansen, J. D. & Nielsen, S. B. The periglacial engine of mountain erosion – Part 2: Modelling large-scale landscape evolution. *Earth Surface Dynamics* **3**, 463–482 (2015).

8. Andersen, J. L., Egholm, D. L., Knudsen, M. F., Jansen, J. D. & Nielsen, S. B. The periglacial engine of mountain erosion – Part 1: Rates of frost cracking and frost creep. *Earth Surface Dynamics* **3**, 447–462 (2015).

9. Aas, K. S. *et al.* Thaw processes in ice-rich permafrost landscapes represented with laterally coupled tiles in a land surface model. *The Cryosphere* **13**, 591–609 (2019).

10. Muster, S., Langer, M., Heim, B., Westermann, S. & Boike, J. Subpixel heterogeneity of ice-wedge polygonal tundra: a multi-scale analysis of land cover and evapotranspiration in the Lena River Delta, Siberia. *Tellus B: Chemical and Physical Meteorology* **64**, 17301 (2012).

11. Ulrich, M., Grosse, G., Strauss, J. & Schirrmeister, L. Quantifying Wedge-Ice Volumes in Yedoma and Thermokarst Basin Deposits. *Permafrost and Periglacial Processes* **25**, 151–161 (2014).

12. Koven, C. D. *et al.* Permafrost carbon-climate feedbacks accelerate global warming. *Proceedings of the National Academy of Sciences* **108**, 14769–14774 (2011).

13. Lawrence, D. M., Slater, A. G. & Swenson, S. C. Simulation of Present-Day and Future Permafrost and Seasonally Frozen Ground Conditions in CCSM4. *Journal of Climate* **25**, 2207–2225 (2012).

14. Slater, A. G. & Lawrence, D. M. Diagnosing Present and Future Permafrost from Climate Models. *Journal of Climate* **26**, 5608–5623 (2013).

15. Koven, C. D., Lawrence, D. M. & Riley, W. J. Permafrost carbon–climate feedback is sensitive to deep soil carbon decomposability but not deep soil nitrogen dynamics. *Proceedings of the National Academy of Sciences* **112**, 3752–3757 (2015).

16. NSIDC. Cryosphere Glossary, frozen ground or permafrost (2020). Https://nsidc.org/cryosphere/glossary-terms/frozen-ground-or-permafrost.

17. Kokelj, S. V. & Jorgenson, M. T. Advances in Thermokarst Research. *Permafrost and Periglacial Processes* **24**, 108–119 (2013).

18. Lee, H., Swenson, S. C., Slater, A. G. & Lawrence, D. M. Effects of excess ground ice on projections of permafrost in a warming climate. *Environmental Research Letters* **9**, 124006 (2014).

19. Westermann, S. *et al.* Simulating the thermal regime and thaw processes of ice-rich permafrost ground with the land-surface model CryoGrid 3. *Geosci. Model Dev.* **9**, 523–546 (2016).

20. Langer, M. *et al.* Rapid degradation of permafrost underneath waterbodies in tundra landscapes—Toward a representation of thermokarst in land surface models. *Journal of Geophysical Research: Earth Surface* **121**, 2446–2470 (2016).

21. Painter, S. L. *et al.* Integrated surface/subsurface permafrost thermal hydrology: Model formulation and proof-of-concept simulations. *Water Resources Research* **52**, 6062–6077 (2016).

22. Abolt, C. J., Young, M. H., Atchley, A. L. & Harp, D. R. Microtopographic control on the ground thermal regime in ice wedge polygons. *The Cryosphere* **12**, 1957–1968 (2018).

23. Schirrmeister, L. *et al.* Periglacial landscape evolution and environmental changes of Arctic lowland areas for the last 60 000 years (western Laptev Sea coast, Cape Mamontov Klyk). *Polar Research* **27**, 249–272 (2008).

24. Fedorov, A. N. *et al.* Permafrost-Landscape Map of the Republic of Sakha (Yakutia) on a Scale 1:1,500,000. *Geosciences* **8**, 465 (2018).

25. Nitze, I., Grosse, G., Jones, B. M., Romanovsky, V. E. & Boike, J. Remote sensing quantifies widespread abundance of permafrost region disturbances across the Arctic and Subarctic. *Nature Communications* **9**, 5423 (2018).

26. Günther, F., Overduin, P. P., Sandakov, A. V., Grosse, G. & Grigoriev, M. N. Short- and long-term thermo-erosion of ice-rich permafrost coasts in the Laptev Sea region. *Biogeosciences* **10**, 4297–4318 (2013).

27. Strauss, J. *et al.* Database of Ice-Rich Yedoma Permafrost (IRYP). *PANGAEA* https://doi.org/10.1594/pangaea.861733 (2016).

28. Obu, J., Westermann, S., Kääb, A. & Bartsch, A. Ground Temperature Map, 2000-2016, Northern Hemisphere Permafrost. *PANGAEA* (2018). Type: Dataset, DOI: 10.1594/PANGAEA.888600.
