# Supplementary material for: Fast response of cold ice-rich permafrost in northeast Siberia to a warming climate
Source: Nat Commun. 2020 May 4;11:2201. doi: 10.1038/s41467-020-15725-8 (PMC7198584; doi:10.1038/s41467-020-15725-8)
Supplement: Supplementary file 3 — Description of Additional Supplementary Files [file 41467_2020_15725_MOESM3_ESM.docx]

Description of Additional Supplementary Files

File: Supplementary Movie 1

Description: Holocene Deposits RCP45 water-logged

File: Supplementary Movie 2

Description: Holocene Deposits RCP45 well-drained

File: Supplementary Movie 3

Description: Holocene Deposits RCP85 water-logged

File: Supplementary Movie 4

Description: Holocene Deposits RCP85 well-drained

File: Supplementary Movie 5

Description: Lake Basins RCP45 water-logged

File: Supplementary Movie 6

Description: Lake Basins RCP45 well-drained

File: Supplementary Movie 7

Description: Lake Basins RCP85 water-logged

File: Supplementary Movie 8

Description: Lake Basins RCP85 well-drained

File: Supplementary Movie 9

Description: Yedoma Deposits RCP45 water-logged

File: Supplementary Movie 10

Description: Yedoma Deposits RCP45 well-drained

File: Supplementary Movie 11

Description: Yedoma Deposits RCP85 water-logged

File: Supplementary Movie 12

Description: Yedoma Deposits RCP85 well-drained
